# Response Modifiers: Tweaking the Immune Response Against Influenza A Virus

**DOI:** 10.3389/fimmu.2019.00809

**Published:** 2019-04-12

**Authors:** Husni Elbahesh, Thomas Gerlach, Giulietta Saletti, Guus F. Rimmelzwaan

**Affiliations:** Research Center for Emerging Infections and Zoonoses, University of Veterinary Medicine (TiHo), Hanover, Germany

**Keywords:** influenza, treatment, response modifiers, antiviral, immune response, immunomodulators

## Abstract

Despite causing pandemics and yearly epidemics that result in significant morbidity and mortality, our arsenal of options to treat influenza A virus (IAV) infections remains limited and is challenged by the virus itself. While vaccination is the preferred intervention strategy against influenza, its efficacy is reduced in the elderly and infants who are most susceptible to severe and/or fatal infections. In addition, antigenic variation of IAV complicates the production of efficacious vaccines. Similarly, effectiveness of currently used antiviral drugs is jeopardized by the development of resistance to these drugs. Like many viruses, IAV is reliant on host factors and signaling-pathways for its replication, which could potentially offer alternative options to treat infections. While host-factors have long been recognized as attractive therapeutic candidates against other viruses, only recently they have been targeted for development as IAV antivirals. Future strategies to combat IAV infections will most likely include approaches that alter host-virus interactions on the one hand or dampen harmful host immune responses on the other, with the use of biological response modifiers (BRMs). In principle, BRMs are biologically active agents including antibodies, small peptides, and/or other (small) molecules that can influence the immune response. BRMs are already being used in the clinic to treat malignancies and autoimmune diseases. Repurposing such agents would allow for accelerated use against severe and potentially fatal IAV infections. In this review, we will address the potential therapeutic use of different BRM classes to modulate the immune response induced after IAV infections.

## Introduction

Influenza viruses (IVs) are responsible for significant morbidity and mortality in the human population with ~500,000 annual deaths worldwide. IVs can cause severe acute respiratory disease especially in high-risk populations like children, the elderly and the immunocompromised. While both influenza A and B viruses (IAV and IBV, respectively) cause annual epidemics, the majority of severe human infections are caused by IAV.

IVs have segmented negative-sense single-stranded RNA genomes. The lack of proof-reading activity of the viral RNA-dependent RNA polymerase (RdRp) and successive replication can lead to the accumulation of nucleotide mutations which drive antigenic drift. In addition, the segmented nature of their genome allows genetic reassortment between IV's to take place, which can produce novel strains that have acquired alternative antigenically distinct hemagglutinin, also known as antigenic shift. Both antigenic drift and antigenic shift contribute to the IV's ability to evade pre-existing host immunity induced by previous infections.

Early recognition and responses to IV infection are largely mediated by innate immune sensors expressed by its primary target, the alveolar epithelial cells (1, 2). Recognition of IVs is mediated by pattern recognition receptors (PRRs) that include Toll like receptors (TLRs), retinoinc acid inducible gene-I (RIG-I), and nucleotide oligomerization domain (NOD)-like receptor family pyrin domain containing 3 (NLRP3); all of which can recognize viral RNAs during various stages of the infection cycle (3–5). Activation of these sensors triggers signaling cascades that lead to the production of interferons as well as pro-inflammatory cytokines and chemokines ultimately resulting in an antiviral state within the surrounding cells/tissue (6). Accordingly, IVs have multiple mechanisms to evade these responses mediated by the viral nonstructural 1 protein (NS1), polymerase basic 1 protein (PB1), polymerase basic 2 protein (PB2), polymerase acidic (PA) and nucleoprotein (NP) [reviewed in van de Sandt et al. (1) and Chen et al. (2)].

In otherwise healthy individuals, IAV infections are mild and the ensuing pro- and anti-inflammatory responses are balanced. In contrast, a “cytokine storm” is typically associated with severe infections including those caused by highly pathogenic IV strains. During a cytokine storm, chemokine and cytokine responses are dysregulated in both intensity and kinetics resulting in excessive damage to the host due to infiltration of inflammatory immune cells. Acute lung injury (ALI) caused by this inflammatory response is typically characterized by significant damage or destruction of the respiratory epithelium leading to acute respiratory distress syndrome (ARDS) (7, 8).

Clinical treatment options for severe influenza virus infections remain limited and relying heavily on the administration of antiviral neuraminidase inhibitors (NAIs) and supportive critical care (9). However, NAIs have not been effective in patients with severe H7N9 or H5N1 infections and there is evidence that fatal outcomes are associated with development of antiviral resistance in patients (10–12). While virus-targeted therapies remain the standard approach, IV's mutability and adaptation to current antivirals has highlighted the need for new therapeutic options that target host factors that regulate IV infections and resulting immune responses. In either approach, the focus is to prevent or limit damage to the lung epithelium due to exaggerated or dysregulated immune cell responses. Biological response modifiers (BRMs) can alter the immune response thereby offering an additional therapeutic approach to treating severe infections. In this review, we highlight several studies that have shown the viability of BRMs as potential treatment options. For clarity, BRMs are categorized based on the type of biological agent (Table 1).

**Table 1:** Biological response modifiers discussed.

**BRM class**	**Target**	**Therapy**	**Activity**	**IAV strain**	**References**
Therapeutic antibodies	HA	MHAA4549A, MEDI8852 and VIS410	– Reduced viral replication. – Improved symptoms of human patients in phase 2 clinical trials	– Circulating seasonal (2015/16) IAV; Seasonal IAV (H3N2) challenge	(13–15)
	ANGPTL4	Anti-ANGPTL4	– Reduced pulmonary tissue leakiness, significantly accelerated lung recovery and improved lung tissue integrity in mice.	– Mouse-adapted laboratory IAV (H1N1)	(16)
	C5a	IFX-1 antibody	– Reduced viral load and virus-induced ALI due to reduced infiltration of lung macrophages and neutrophils in IAV-infected African green monkeys.	– Highly-pathogenic avian IAV (H7N9)	(17)
	TRAIL	Anti-Trail	– Increased survival rate following IAV infections in mouse studies.	– Mouse-adapted laboratory IAV (H1N1 and its derivative H3N2)	(18, 19)
	TNFα	Anti-TNFα	– Reduced disease burden in mouse studies. – No effect on viral replication.	– Mouse-adapted laboratory IAV (H1N1-derived H3N2)	(20)
Therapeutic peptides	AMP	LL-37	– Reduced morbidity and mortality to similar levels as zanamivir in mice.	– Mouse-adapted laboratory IAV (H1N1)	(21)
	Influenza A virus	TAT-Kα2	– Complete protection of infected mice. – Direct virocidal activity.	– Highly-pathogenic avian IAV (H5N1)	(22)
Therapeutic small molecules	JNK1/JNK2	SP600125, AS601245	– Reduced levels of pro-inflammatory cytokines and reduced viral titers in mice.	– Highly-pathogenic avian IAV (H7N7); 2009 pandemic IAV (H1N1)	(23, 24)
	p38	SB202190, SB203580	– Mice were protected from lethal H5N1 infection exhibiting reduced mortality and pro-inflammatory responses.	– Highly-pathogenic avian IAV (H5N1)	(25)
	MEK	CI-1040	– Reduced lung viral load and mortality of IAV-infected mice.	– 2009 pandemic IAV (H1N1)	(26)
	NFkB	SC75741	– Reduced mortality and morbidity in mice following highly pathogenic IAV infections. – Similar results prophylactically.	– Highly-pathogenic avian IAV (H7N7 and H5N1)	(27)
	GRK2	Paroxetine	– Reduced viral load. – No effect on mortality in IAV-infected mice.	– 2009 Pandemic IAV (H1N1)	(28)
	SphK1/SphK2	SK-1I, SK-2I, and Pan-SKI	– Prolonged survival of mice following lethal IAV infection.	– Mouse-adapted laboratory IAV (H1N1)	(29)
	PAR1	SCH79797	– Increased survival and a decrease in inflammatory responses in H5N1 or H1N1 infected mice. – Similar effect when administered 48–72 h after infection.	– Mouse-adapted IAVs (H1N1 and H3N2); Oseltamivir-resistant 2009 pandemic IAV isolate (H1N1); highly-pathogenic avian IAV (H5N1)	(30)
	PPARα/PPARγ	Gemfibrozil (PPARα), Pioglitazone (PPARγ)	– Improved symptoms and increased survival of IAV infected mice. ed survival after H1N1 or H5N1 mouse infections.	– 1957 Pandemic IAV (H2N2); mouse-adapted laboratory IAV (H1N1); 2009 pandemic IAV (H1N1)	(31–33)

## Biological Response Modifiers

### Therapeutic Antibodies

IAV infections and some vaccines elicit broadly-neutralizing antibodies (Abs) that target the viral HA-stem. However, their abundance and immune-subdominance is overshadowed by Abs targeting the HA-head domain. The effectiveness of these HA-stem Abs against a broad range of IAV subtypes, makes them an attractive target not only for vaccine development but also as antivirals. Indeed, several HA-stem specific human monoclonal Abs are now being evaluated in clinical trials [reviewed in Davidson (34)]. MHAA4549A, MEDI8852, and VIS410 are human monoclonal Abs that have been shown to control viral replication and improve symptoms of human patients in phase 2 clinical trials (13–15).

While virus-specific Abs aim to reduce antigenic load, Abs to host targets aim at limiting the secondary wave of cytokines and reduce prolonged damaging cellular infiltration during severe infections. Host-target directed antibodies have been utilized to target key regulators of this inflammatory wave and could potentially be used to dampen these overt responses.

Angiopoietin-like 4 (ANGPTL4) is a soluble angiogenic-regulating protein. Following proteolytic cleavage, the C-terminal portion (cANGPTL4) is involved in integrin-dependent wound repair and can regulate vascular permeability (35, 36). ANGPTL4 was significantly elevated in lung biopsies from IAV-induced pneumonia patients (16). In mouse studies, neutralizing anti-ANGPTL4 Abs reduced pulmonary tissue leakiness significantly accelerating lung recovery and improved lung tissue integrity (16).

Neutrophil infiltration into the alveolar space occurs within 1 day following IAV infections (37). Neutrophil extracellular traps (NETs) released during IAV-induced pneumonia into the alveolar space caused alveolar damage (38). The complement protein C5a was shown to induce NETs release and administration of anti-C5a Abs (IFX-1) reduced H7N9-induced ALI due to reduced infiltration of lung macrophages and neutrophils as well as reduction of viral load in African green monkeys (17, 39).

Tumor necrosis factor alpha (TNFα) is a key cytokine for controlling severe IAV infections. It regulates two main antiviral functions: the induction of (i) the NFkB pathway, which ultimately controls expression of several inflammatory cytokines and (ii) apoptosis through multiple signaling cascades (40, 41). TNF upregulation during IAV infections correlates with infection severity, especially following highly pathogenic IAV-infections (42–44). Mice treated with anti-TNF Abs showed reduced disease burden; however, the authors of that study reported no effect on viral replication (20).

TNF-related apoptosis inducing ligand (TRAIL) can trigger apoptosis in IAV-infected cells. IAV-infected human epithelial cells are sensitized to TRAIL-mediated apoptosis while peripheral blood mononuclear cells upregulate TRAIL expression. Moreover, administration of monoclonal Abs against TRAIL increases survival rate following IAV infections in mouse studies (18, 19).

### Therapeutic Peptides

Antimicrobial peptides (AMPs) are host proteins that have direct antibacterial and antiviral activities and can modulate immune responses to infections. While the literature is largely focused on the antibacterial aspects of AMPs, several studies have highlighted the antiviral potential of AMPs against several viruses including IVs [reviewed in Hsieh and Hartshorn (45) and Albericio and Kruger (46)]. LL-37 is a human cathelicidin derived AMP that is found predominantly in neutrophils and its expression can also be induced in epithelial cells and macrophages (47). Aerosol administration of either human LL-37 or its mouse counterpart mCRAMP led to reduced morbidity and mortality to similar levels as the neuraminidase inhibitor zanamivir that is used for the treatment of human influenza patients (21).

Both cellular and viral FADD-like IL-1β-converting enzyme-inhibitory protein (cFLIP and vFLIP, respectively) protect cells from death receptor mediated apoptosis. Kα2 is a vFLIP-derived peptide that consists of 10 amino acids from the α2 helix of the Kaposi's sarcoma herpes virus (KSHV) death effector domain 1 protein. A synthetic version of this peptide, TAT-Kα2, was generated by fusing Kα2 to a portion of the HIV TAT protein (22, 48). In mouse challenge studies, intranasal administration of TAT-Kα2 at the time of infection with highly pathogenic avian H5N1 virus resulted in protection of the treated mice. No replicating virus was detected in the lungs at either 3 or 5 days after infection suggesting complete protection from infection (22). It should be noted that this effect is largely due to direct destabilization of the virions by the TAT-Kα2 peptide and it is likely that infection in treated mice was not established; the efficacy of this AMP has not been determined during an established infection and warrants further investigation.

### Therapeutic Small Molecules

Host kinases regulate not only IAV entry and replication but also initiate antiviral signaling cascades that regulate expression of pro-inflammatory chemokines and cytokines during infections and present viable targets for intervention (24, 49–58).

IAV infection has been shown to upregulate c-Jun N-terminal kinases 1 and 2 (JNK1/JNK2). These kinases directly regulate the induction of pro-inflammatory responses. IAV-induced JNK1/JNK2 activation mediates production of chemokines and cytokines including TNF-α, interferon β (IFN-β), and interleukin 6 (IL-6) (24). *In vivo* inhibition of JNK1/JNK2 resulted in reduced levels of pro-inflammatory cytokines and reduced viral titers (23, 24).

The mitogen activated protein kinase (MAPK), p38, regulates viral entry and replication (55, 59). Furthermore, p38 regulates IFN stimulated gene (ISG) gene expression and ultimately cytokine production via STAT1 phosphorylation (25). Using either of two specific p38 inhibitors (SB 202190 or SB 203580), mice were protected from lethal H5N1 infection exhibiting reduced mortality and pro-inflammatory responses (25). Activation of another MAPK, MEK, is required for efficient IAV replication and its inhibition results in viral ribonucleoprotein (vRNP) retention and reduced titers of progeny virus (26, 60, 61). Importantly, treatment of mice with the clinically approved MEK inhibitor (CI-1040) showed reduced lung viral load and mortality of mice following infection with a lethal dose of pandemic H1N1 IAV; interestingly, this inhibitor significantly out-performed the clinically recommended oseltamivir in these studies (26).

Another central regulator of immune responses at the epithelium as well as immune cells is the NF-κB signaling pathway. Accordingly, IAV has evolved several mechanisms to modulate this pathway to counteract antiviral responses including directly targeting the IkB kinase (IKK) (62, 63). SC75741 is a potent NFkB inhibitor that functions by reducing the ability of the p65 subunit of the NFkB complex to bind DNA; thereby limiting its transcription-regulating functions (64, 65). *In vivo* administration of SC75741 at 4 days after lethal infection with either H5N1 or H7N7 avian viruses resulted in significant protection with most mice surviving and showing little to no clinical symptoms; similar results were obtained by prophylactic administration (27).

G-protein coupled receptor kinase 2 (GRK2) is best known for its phosphorylation of GPCRs in cardiac tissue resulting in recruitment of β-arrestin to facilitate rapid receptor internalization and lysozomal degradation (66). Recent phosphoproteomic studies identified GRK2 as a potentially proviral host protein for IAV that plays a major role in virion uncoating (28). Although *in vivo* inhibition of GRK2 using paroxetine led to a significant reduction in upper respiratory tract viral load and to a modest reduction in lower respiratory tract titers at 4 days post infection, this inhibition was not protective from lethal infections (28). However, it is possible that the route of administration (intraperitoneal vs. intranasal) and dosing regimen influenced the results.

Sphingosin kinases (SphK) are lipid kinases that mediate conversion of sphingosine to bioactive lipid sphingosine 1-phosphate (S1P) (67), a known modulator of central apoptotic pathways (68). IAV infections leads to increased expression and activation of SphK1 and SphK2 (29) and *in vitro* inhibition of SphK1 was shown to decrease IAV RNA synthesis via suppression of NFkB activation (69). Treatment of mice with specific inhibitors to either SphK1 or SphK2 or a pan-SphK inhibitor led to prolonged survival of mice following lethal IAV infection (29).

Peroxisome proliferator-activated receptors (PPARα, PPARβ, and PPARγ) regulate metabolic homeostasis and are important mediators of the inflammatory response. Several PPAR agonists have been investigated for efficacy during IAV infections with varying results. Gemfibrozil (PPARα agonist) not only improved symptoms when administered 4 days after infections with an H2N2 virus, but also increased survival of IAV infected mice (31). Prophylactic treatment of H1N1-infected mice with pioglitazone (PPARγ agonist) resulted in increased survival (32). Combined activation of PPARγ and its downstream target AMPK improved survival of mice infected with pandemic IAV strains (33).

Protease activated receptor (PARs) link protease activity to inflammatory cellular responses (70). PAR1 expression is upregulated in the mouse airways following IAV infections (71). Intranasal administration of a PAR1 antagonist (SCH79797) at the time of infection with various IAV strains including highly pathogenic avian H5N1 and pandemic H1N1 viruses led to increased survival and a decrease in inflammatory responses. Moreover, this effect was also observed when SCH79797 was administered 48–72 h after infection (30).

The use of statins, angiotensin II receptor blockers (ARBs) and angiotensin converting enzyme inhibitors (ACEi) has been proposed to regulate the IAV-induced cytokine storm in severe infections (72, 73). Retrospective studies conducted separately in Mexico, Netherlands, UK and USA reported an association of reduced IAV-related pneumonia and lower case fatality due to lower respiratory tract IAV infections with statin treatment (74–77). However, this association was contested in two additional studies that found no benefit of statin treatment on IAV-induced disease burden (78, 79). This uncertainty regarding the IAV therapeutic potential of these widely used compounds warrants further investigations at the basic science level and in clinical trials.

## Perspectives and Future Directions

**Figure 1:**
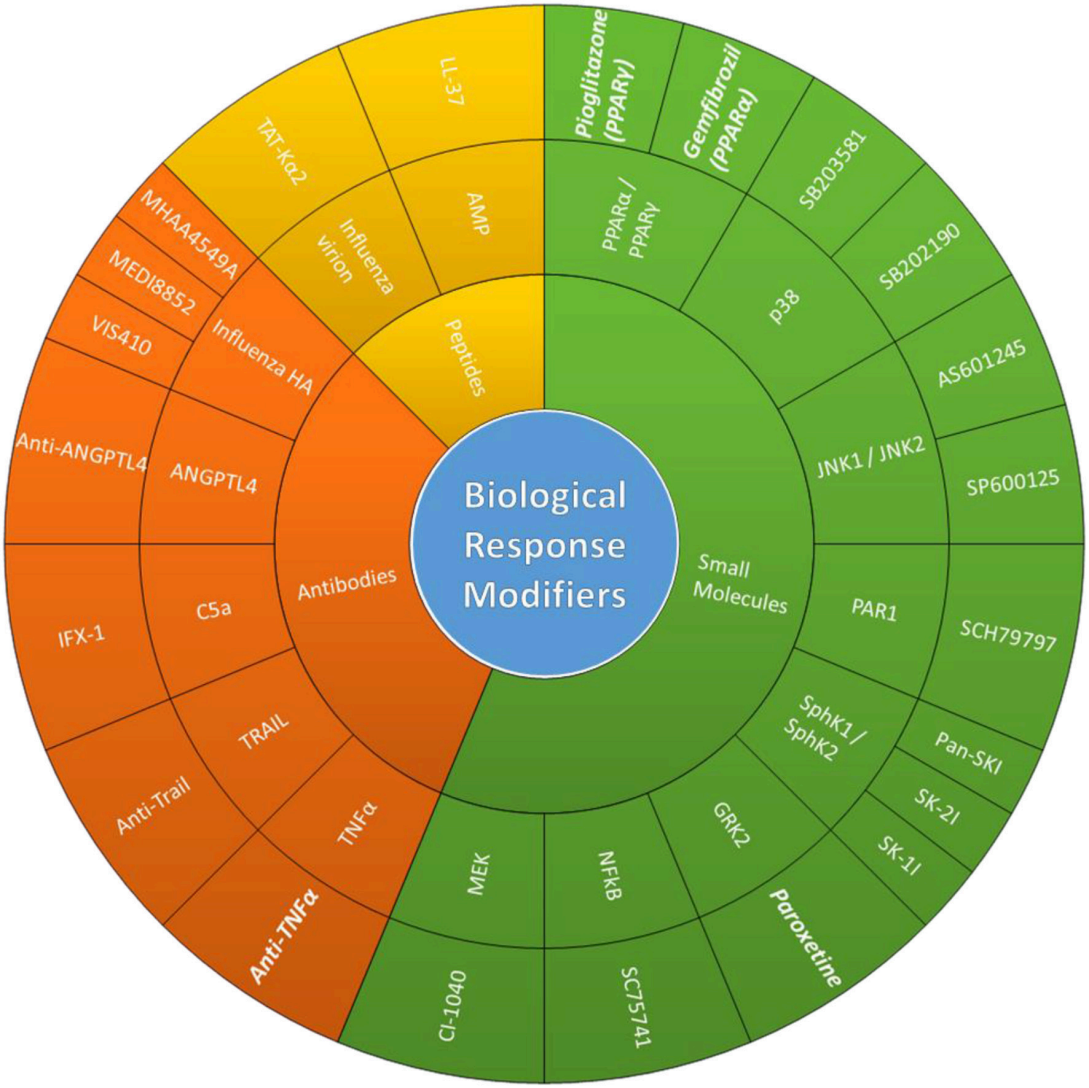
Biological response modifiers (BRMs). Potentially therapeutic BRMs that have shown antiviral/immunomodulatory effects during *in vivo* IV infections. Schematic organizing BRMs based on BRM class, host/virus targets, compounds used in cited studies (from innermost to outermost ring). FDA-approved BRMs cited in this review are in bold and italics.

The continuous accumulation of adaptive mutations and the introduction of novel viruses in the human population continue to pose a threat to public health, especially to individuals at high risk to influenza. The emergence of strains resistant to existing classes of antiviral drugs and reduced vaccine effectiveness highlights the need for the development of alternative intervention strategies. Therefore, therapeutic approaches that can diminish the potential for drug-resistance while being effective against multiple IAV subtypes/strains are highly desirable. Targeting host cell factors meets these criteria and is more likely to avoid overtly robust immune responses thereby reducing disease severity and improve patient outcome (Figure 1).

A large effort has been made in recent years to identify host proteins to serve as intervention targets against IV infections. Several genetic and proteomic screens have identified several promising hits with potential roles in the IV replication cycle (80–90). In addition to these genome-wide screens, viral and host protein interactions can be mapped into networks that can also be used to identify host factors critical for IV replication (91, 92). Interestingly, meta-analysis of some these studies shows limited overlap in the genes/proteins identified as required host factors (87, 93–95). This is likely due to study-specific variations in IV types/strains and cell-lines used, inclusion/exclusion criteria, limited hit-validations and methods used to “knock-down/out” these genes.

Local microenvironment within a given tissue can dictate the quality and intensity of an immune response. Inhibition or activation of critical signaling pathways expressed in both respiratory tract epithelial and immune cells by BRMs can have opposite and unintended consequences. As discussed above, TRAIL regulates immune cell-mediated apoptosis of infected cells and several studies have shown that blocking TRAIL signaling by genomic deletion or depletion by monoclonal antibody administration can improve infection outcome in IAV-infected mice. Indeed inhibition of TRAIL signaling in alveolar macrophages and other monocytes limits their ability to induce apoptosis in alveolar cells, prevents lung tissue damage and promotes survival (19, 96, 97). However, CD8+ T cells from TRAIL−/− mice are less able to protect mice from severe infections, consistent with impaired TRAIL-mediated effector functions of CD8+ T cells (18). Similarly, opposing beneficial and detrimental outcomes have also been observed in studies using Bcl-2 inhibitors to treat IAV infections (98, 99).

BRM delivery should be guided by immune system “compartmentalization” to ensure they elicit balanced immune responses. Ideally, mucosal delivery deposits BRMs that reduce viral titers at the site of IAV replication; however, systemic delivery of certain BRMs might be required to dampen dysregulated responses. This not only depends on the BRMs used but also on the timing of their administration. Moreover, the duration of treatment with BRMs must be considered because sustained inhibition of certain inflammatory responses can result in an immune status that increases susceptibility to secondary opportunistic infections.

Repurposing of clinically approved drugs could potentially be used as BRMs for the treatment of severe IAV infectious and should be explored (86, 89, 90). Considering that susceptibility to severe IAV infections is influenced by host genetics and host-specific immune responses, selection of therapeutic BRMs should be carried out using *in vivo* model systems that are representative of the immune status spectrum and underlying conditions of high-risk influenza patients (young, immunocompromised, non-naive, obese, pregnant, or aged). Using these model systems will increase the likelihood of identifying BRMs with clinically relevant antiviral and immunomodulatory potentials.

## Author Contributions

HE, TG, GS, and GR conceptualized and composed the manuscript. GR and HE oversaw all aspects of the manuscript preparation.

### Conflict of Interest Statement

The authors declare that the research was conducted in the absence of any commercial or financial relationships that could be construed as a potential conflict of interest.
